# Point of care gastric ultrasound to predict aspiration in patients undergoing urgent endotracheal intubation in the emergency medicine department

**DOI:** 10.1186/s12873-023-00881-z

**Published:** 2023-09-21

**Authors:** Reshma Asokan, Bharat Bhushan Bhardwaj, Naman Agrawal, Udit Chauhan, Aadya Pillai, Takshak Shankar, D. J. Lalneiruol, Himanshi Baid, Hannah Chawang, Sanket Mukeshkumar Patel

**Affiliations:** 1 Department of Emergency Medicine, All India Institute of Medical Sciences Rishikesh, Rishikesh, 249203 Uttarakhand India; 2 Department of Trauma and Emergency, All India Institute of Medical Sciences Raipur, Raipur, India; 3 Department of Radiodiagnosis, All India Institute of Medical Sciences Rishikesh, Rishikesh, 249203 Uttarakhand India; 4 Department of Emergency Medicine, Nootan Medical College and Research Centre, Visnagar, Gujarat India

**Keywords:** Aspiration, Emergency department, PoCUS, Gastric ultrasound, Antral parameters, Diagnostic accuracy

## Abstract

**Background:**

One significant cause of morbidity and mortality in patients undergoing endotracheal intubation is the aspiration of gastric contents. Its prevalence is more in the emergency than in elective settings. Point-of-care gastric ultrasound (GUS) is a non-invasive bedside ultrasonogram that provides both qualitative and quantitative information about the stomach contents. The diagnostic accuracy of GUS in terms of gastric parameters (measured antral diameters, antral cross-sectional area, and calculated gastric volume) to predict aspiration is yet unknown. We aim to determine this in the patients undergoing urgent emergency intubation (UEI) in the emergency department.

**Methodology:**

A prospective observational study was conducted at the emergency department of a tertiary healthcare center in India. Patients requiring UEI were identified and a bedside gastric ultrasound was done in the right lateral decubitus position using low frequency curved array probe. The qualitative data and the antral diameters (anteroposterior and craniocaudal) were assessed. The patient's clinical parameters and history regarding the last meal were noted. The cross-sectional area of gastric antrum was calculated using CSA = (AP × CC) π/4. The gastric volume is estimated using Perla's formula: GV = 27.0 + 14.6(RLD CSA) –1.28(age).

**Results:**

A hundred patients requiring urgent endotracheal intubation were enrolled in the study. Visible aspiration was more in participants with a distended gastric status (χ2 = 16.880, *p* =  < 0.001). The median gastric volume in the patients who aspirated was 146.37 mL, and it ranged from 111.59 mL-201.01 mL. Using ROC analysis, a cut-off of CC diameter ≥ 2.35 cm (sensitivity 88%, specificity 91%) and AP diameter ≥ 5.15 cm (sensitivity 88%, specificity 87%) predicts aspiration. A calculated USG CSA cut-off ≥ 9.27cm^2^ (sensitivity 100%, specificity 87%) and an USG gastric volume ≥ 111.594 mL (sensitivity 100%, a specificity 92%) predicts aspiration.

**Conclusion:**

Point-of-care gastric ultrasound is an useful non-invasive bedside tool for risk stratification for aspiration in busy emergency rooms. We present threshold gastric antral parameters that can be used to predict aspiration along with its diagnostic accuracy. This can help the treating ED physician take adequate precautions, decide on intubation techniques and treatment modifications to aid in better patient management.

## Background

Airway management is of utmost priority in the emergency room. It is considered a high-risk procedure for numerous reasons, including limited time for preparation, the unstable condition of the patient, and the urgency of the situation [1]. One of the significant causes of morbidity and mortality in patients undergoing endotracheal intubation is gastric aspiration [2, 3]. Aspiration is the inhalation of either oral, pharyngeal or gastric contents into the lower airways [4]. During intubation, the physiological mechanisms that protect the airway against aspiration, like the tone of the lower esophageal sphincter and upper airway reflexes, are impeded by the induction and paralytic agents [5, 6].

Aspiration risk is directly linked to the prandial status of the patient. Patients with residual gastric volume (GV) of more than 1.5 ml/kg are considered to have significant aspiration risk even though the exact threshold is not yet known [7–9]. Paracetamol absorption, radiolabeled diet, scintigraphy, and aspiration of gastric contents are invasive methods to determine residual GV [10–12]. In the ED, clinical history of meal intake is the only tool available to assess gastric aspiration risk. A minimum of 2 h of fasting after clear fluids, 6 h for a light meal, and at least 8 h after a full meal with high calorie or fat content are the current nil per oral (NPO) recommendations by the American Society of Anesthesiologists [13]. It has a doubtful advantage in patients with comorbidities like diabetes, gastroparesis, renal impairment, or in the critically ill, whose gastric emptying time is longer owing to many factors, including age, diagnosis, and medications [14–16]. Bouvet et al. (2017) found that the prevalence of full stomach was 5% in elective & 56% in emergency surgical patients. Inadequate risk assessment and the unavailability of reliable diagnostic tools can cause poor patient outcomes [17]. Hence, there is a need for a bedside non-invasive test.

Point-of-care gastric ultrasound (GUS) is an emerging radiographic paradigm that is reliable, accurate, brief, and repeatable [18–20]. It provides both qualitative and quantitative information about the stomach contents. Bedside GUS aids in acute care to assess individual risk of aspiration in a condition of clinical uncertainty [21]. The CSA of the gastric antrum can be calculated using the formula, CSA = (AP × CC) x π/4, where AP is the anteroposterior diameter and CC is the craniocaudal diameter. It can also be measured using a free tracing tool in the USG machine. GV can be calculated using the Perlas formula, GV = 27.0 + 14.6 × RLD CSA – 1.28 × age. This formula applies to adults and non-pregnant subjects whose body mass index (BMI) is less than 40 kg m^−2^. It is accurate up to a predicted volume of 500 mL [22].

But the diagnostic accuracy of GUS to detect a full stomach and predict the incidence of aspiration (i.e., the sensitivity, specificity, and positive and negative predictive values) remains to be studied. This study aims to determine the diagnostic accuracy of each antral parameter (AP, CC, CSA, GV) in predicting the risk of aspiration in patients undergoing urgent emergency intubation (UEI) in the emergency department.

## Methods

### Study design and settings

**Fig. 1:**
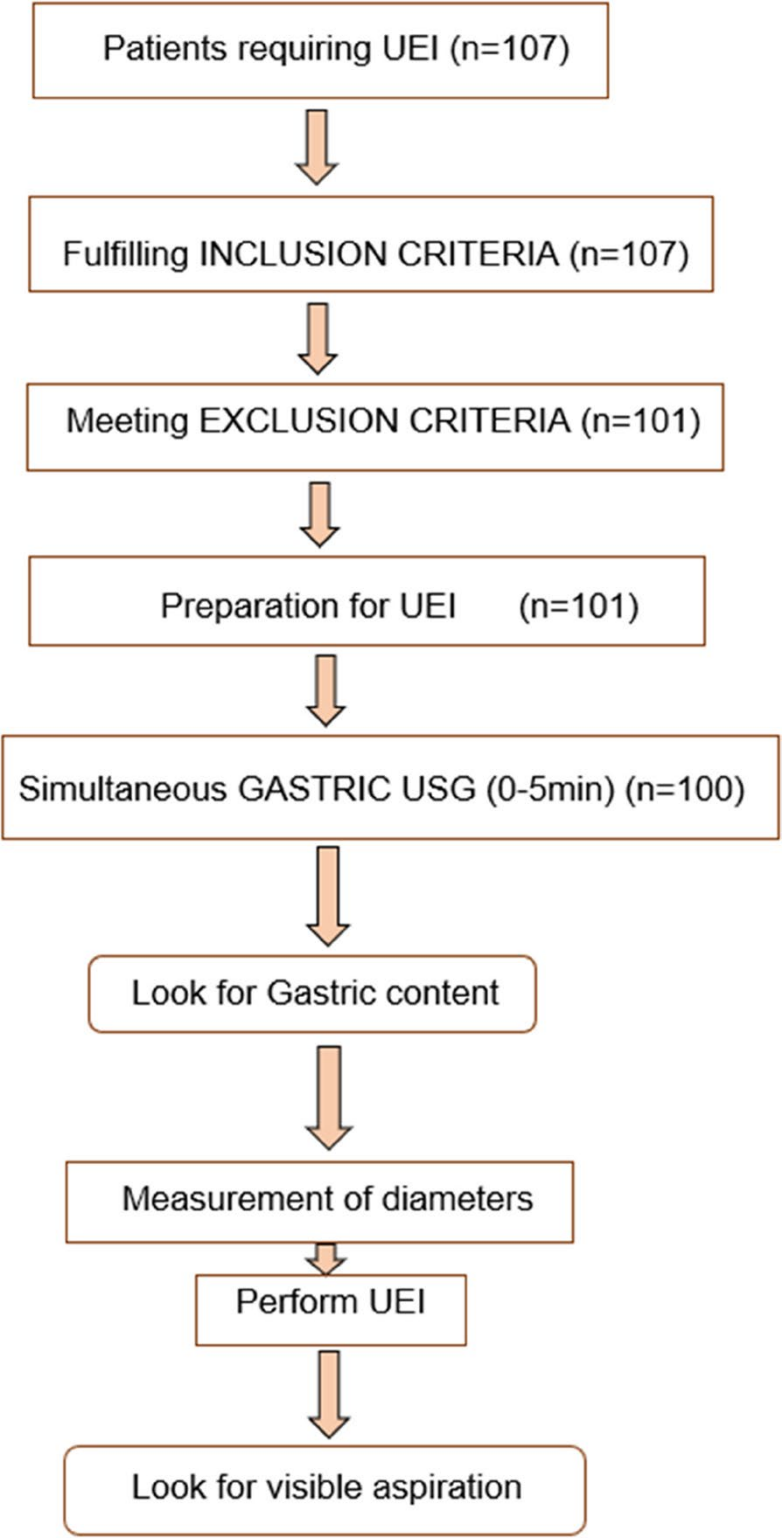
Study flow

A prospective observational study was performed in the Emergency Department, All India Institute of Medical Science, Rishikesh, after approval from the Ethics Committee of the university. The study was conducted over a period from April 2020 to October 2021. Study flow is shown in Fig. 1.

#### Inclusion criteria

1. Age > 18 years.
2. Patients undergoing emergency endotracheal intubation.

#### Exclusion criteria

1. History suggestive of intestinal obstruction or gastric outlet obstruction.
2. Known pregnancy.
3. Morbid obesity.
4. History of gastric/esophageal surgery.
5. Patients in cardiac or respiratory arrest or any patients requiring crash intubation.
6. Patients with a gastric or duodenal tube in situ.
7. Patients/guardians not consenting to take part in the study.
8. Delay of more than 5 min in performing USG due to any cause.

### Sample size

The sample size for the study was assumed based on the incidence of aspiration after emergency intubation by Schwartz et al. who reported the proportion of subjects who had aspiration as 4% [23, 24].

The sample size was calculated according to the formula given by Lemeshow et al., 1990 [25]:

$$\mathrm{Sample size},\mathrm{ N}=\frac{{\left({z}_{1-\left(\alpha /2\right)}\right)}^{2}\times p \times \left(1-p\right)}{{\delta }^{2}}$$

Proportion of subjects with Aspiration, *p* = 0.04 (4%).

Precision, δ = 0.04 (4%).

Type I error, α = 0.05 (5%), ${z}_{\left(1-\alpha /2\right)}$ = 1.96; Beta = 20%, Power: 80%

Based on the formula and values given above:

$$\mathrm{N}=[{1.96}^{2}\mathrm{ x }0.04\mathrm{ x }(1-0.04)]/{0.04}^{2}=91.5\approx 92$$

Thus, 95% confidence interval, the proposed sample size for the study is 92. We expected to include at least 100 members as participants.

### Clinical evaluation

Patients requiring urgent endotracheal intubation who fulfilled the inclusion criteria were promptly identified. The researcher collected detailed history, demographic data, and clinical parameters of the patient at the time of admission after informed consent. Relevant history regarding the last meal, the quantity of the last meal, and the nature (whether liquid or solid) were noted.

### Ultrasound protocol

**Fig. 2:**
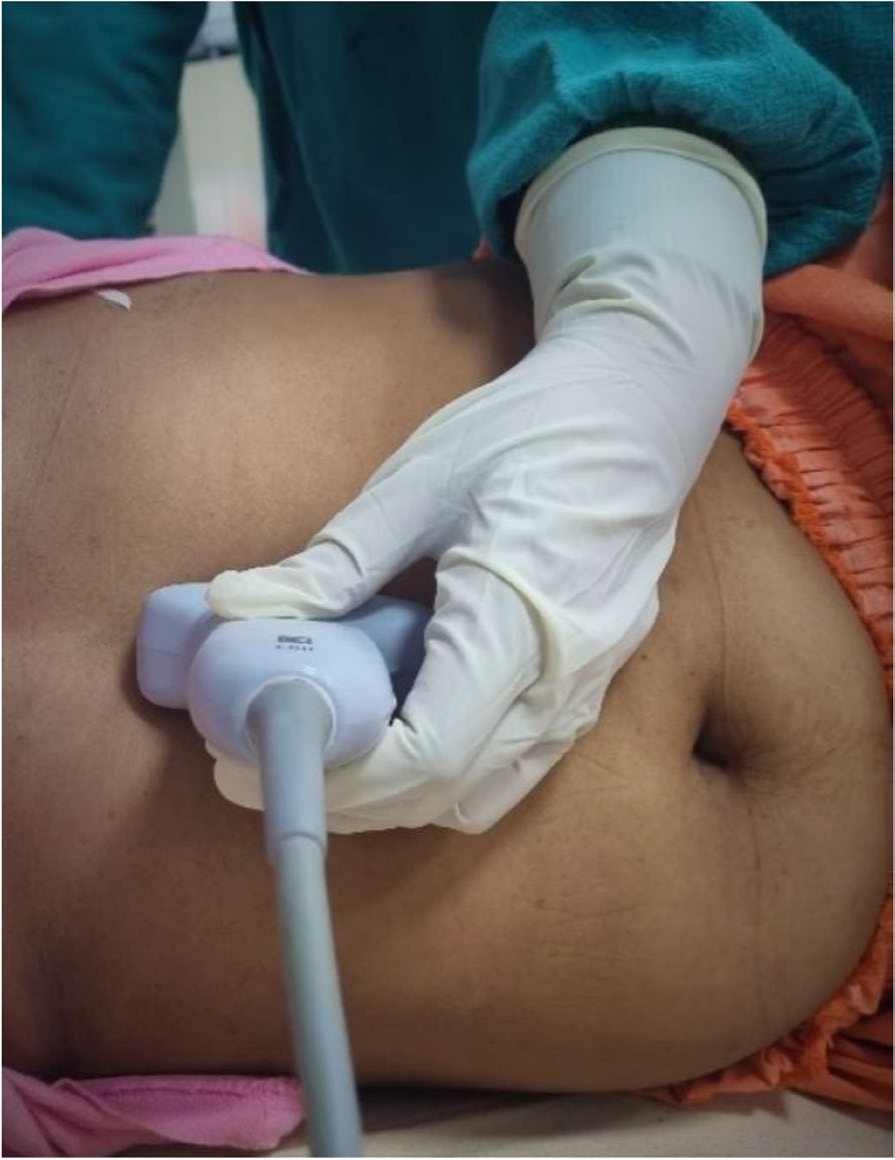
Patient in RLD position and USG done in epigastric sagittal plane

For calculating the gastric volume from gastric diameters using the Perlas formula (GV = 27.0 + 14.6 × RLD CSA – 1.28 × age), the patient had to be kept in the right lateral decubitus (RLD) position [22]. Logroll with cervical inline stabilization was done in trauma patients. A curved array low-frequency USG probe (2–5 MHz) is used. Ultrasound was done by an emergency physician trained in gastric ultrasound (Fig. 2).

**Fig. 3:**
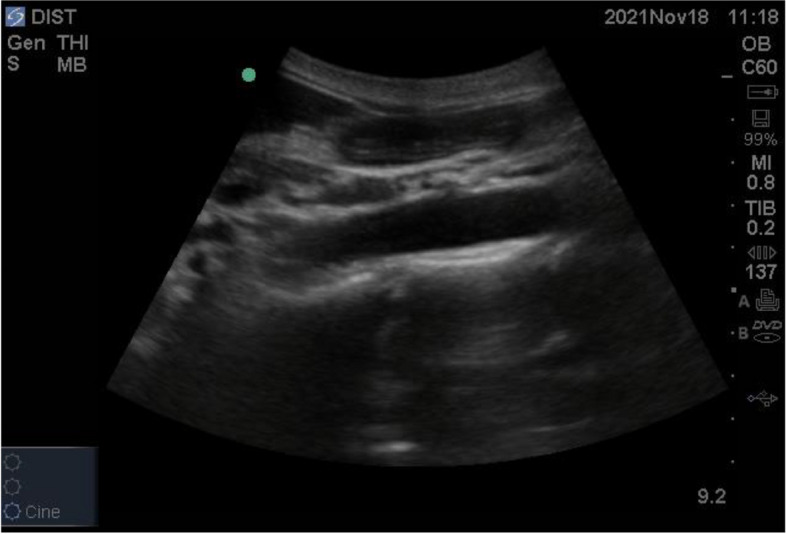
The antral walls juxtaposed and oval signifying empty

**Fig. 4:**
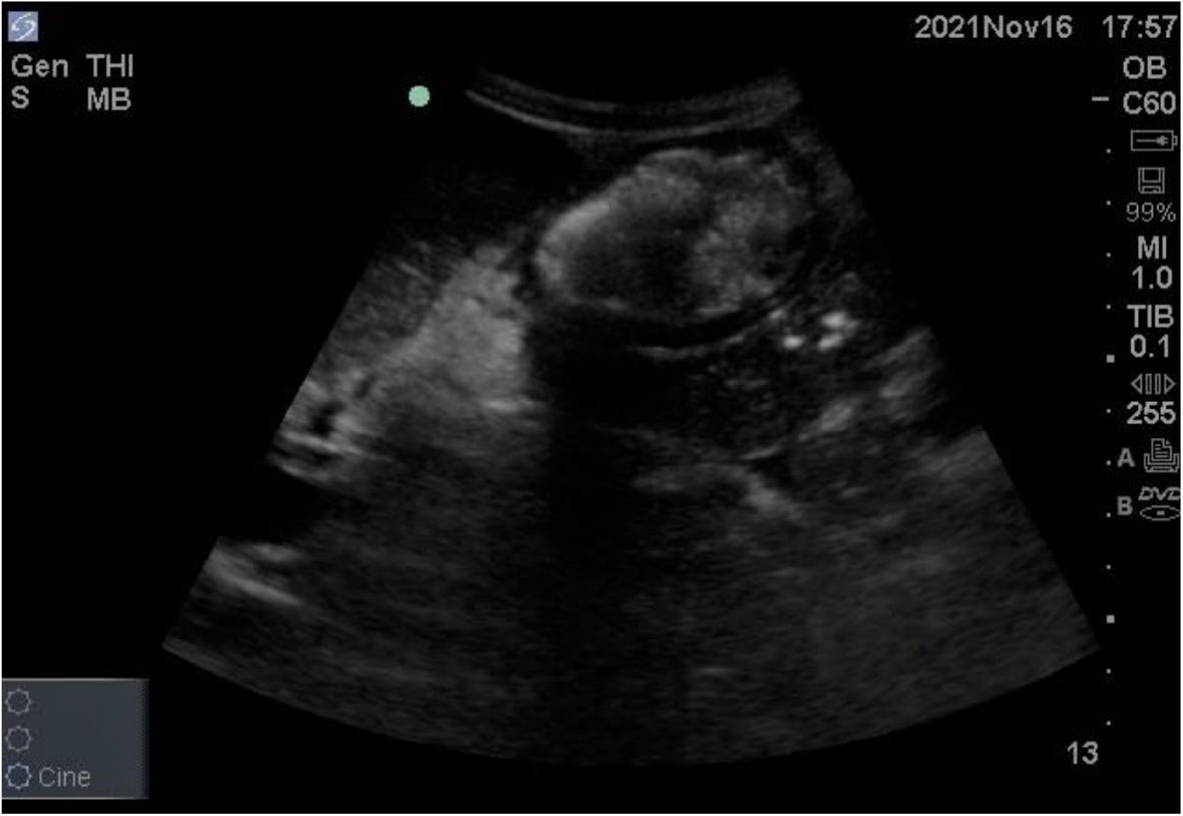
The antrum rounded signifying distended

The most reliably identified part of the stomach is the gastric antrum because of its location and its unique appearance. It lies superficial in the epigastric area inferior to the xiphoid and above the umbilicus and is imaged in a sagittal plane in the RLD position [26]. It appears as a thick hollow organ with a multi-layered wall just below the left lobe of the liver and anterior to the body of the pancreas. The Inferior vena cava and the aorta lie posterior to the antrum. The gastric status can be noted as empty or distended. It is empty when the antral walls are juxtaposed and oval (Fig. 3). It is distended when any stomach content is present and appears rounded [27] (Fig. 4).

**Fig. 5:**
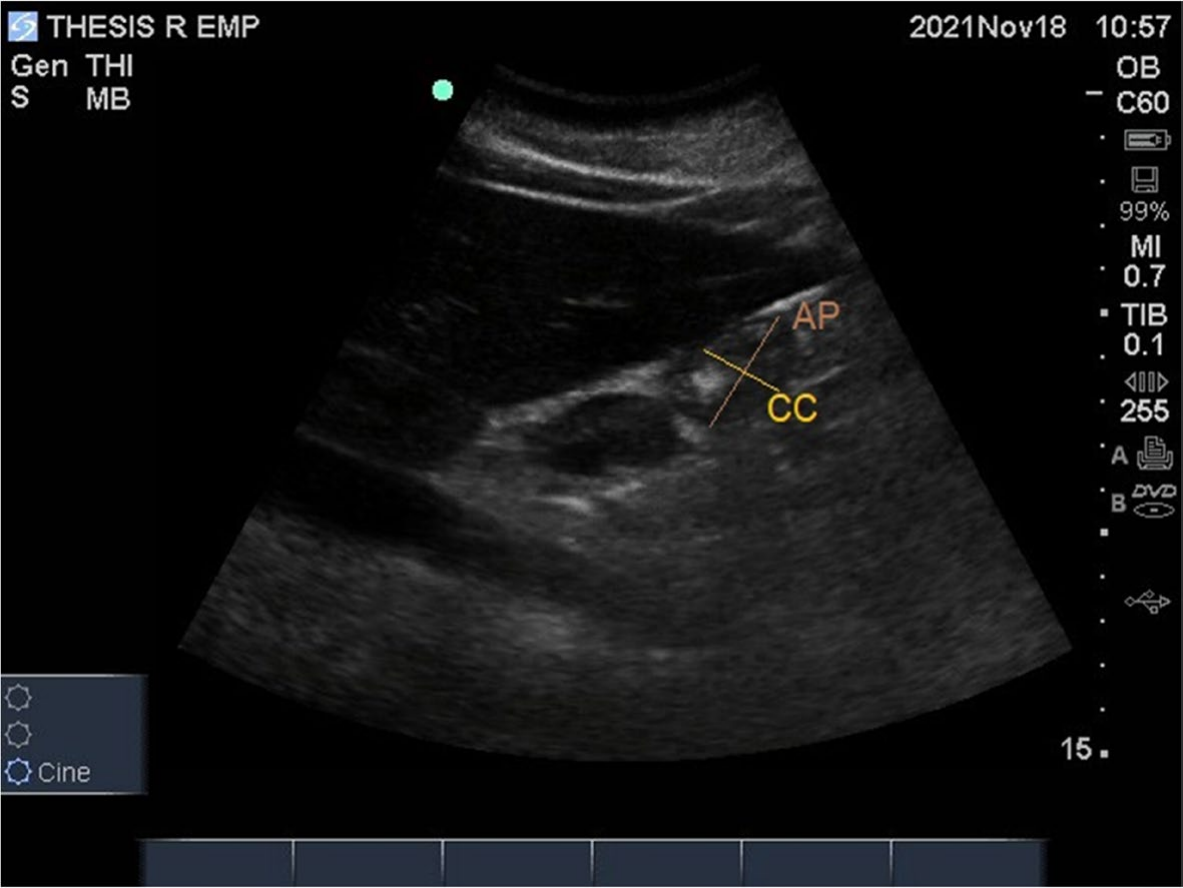
USG gastric antral analysis measuring AP and CC diameters

For quantitative evaluation, the plane at the level of the aorta is the standardized landmark. Two perpendicular diameters, the AP and the CC diameters are measured from serosa to serosa between the peristalsis when the antrum is at rest [28] (Fig. 5). The USG analysis was done in under 5 min while preparing for UEI, not interfering with the course of treatment.

The UEI was carried out by the professional team as per institutional protocol. Any visual aspiration of gastric content seen as any regurgitation into the oropharynx was noted during intubation by the person who intubates. The assessors who decided if an aspiration event occurred were blinded to the results of the ultrasound obtained. If regurgitation is visualized, it is an aspiration present. Any unseen aspiration was taken as no aspiration. The development of clinical or radiographic signs of aspiration pneumonia later during hospital stay was not taken into consideration in the study [29].

### Data collection

The USG analysis was done by Emergency medicine physicians who had received training in GUS for 2 months. The detailed history, demographic data, and clinical parameters of the patient at the time of admission were recorded. The gastric diameters, the AP and the CC diameters were measured using ultrasound. The cross-sectional area, CSA is calculated using CSA = (AP × CC) π/4. The gastric volume, GV is estimated from the calculated CSA using Perla’s formula: GV = 27.0 + 14.6(RLD CSA) – 1.28(age). The negative values in obtained of GV were considered zero [22].

### Statistical analysis

- Data was recorded in MS Excel spreadsheet program. SPSS v23 (IBM Corp.) [30] will be used for data analysis.
- Descriptive statistics was elaborated in the form of means/standard deviations and medians/IQRs for continuous variables, and frequencies and percentages for categorical variables. Normality for continuous data will be checked using Shapiro–Wilk Test.
- Association between two categorical variables was explored using Chi-squared test. In case the expected frequency in the contingency tables will be found to be < 5 for > 25% of the cells, Fisher’s Exact test will be used instead.
- Association between variables where one is continuous and one is categorical was explored using independent sample ‘t’ test when the categorical variable has two categories, and One-Way ANOVA when it has more than 2 categories. If data was found to be non-normally distributed, appropriate non-parametric tests in the form of Wilcoxon Mann–Whitney U Test/Kruskal Wallis test was used for these comparisons.
- Linear correlation between two continuous variables was explored using Pearson’s correlation (if the data will be normally distributed) and Spearman’s correlation (for non-normally distributed data).
- Statistical significance was kept at *p* < 0.05
- Receiver operating characteristic (ROC) curve analysis was done to study the diagnostic accuracy of parameters in predicting aspiration. The results were represented as sensitivity, specificity, PPV, NPV and likelihood ratios, with a confidence interval of 95%

## Results

A prospective observational study was conducted one hundred patients who came to the emergency department of the All India Institute of Medical Sciences, Rishikesh after fulfilling the inclusion and exclusion criteria. We aimed for a data of 100 participants during study period. The main baseline characteristics of the study population are shown in Table 1. A total of 107 patients presenting to ED requiring urgent endotracheal intubation were screened sequentially. Out of this, 3 people required crash intubation, 2 were suspected of having intestinal obstruction and 1 was pregnant. In 1 patient, simultaneous USG analysis of antrum could not be done in 5 min as the machine was in use for another patient in the emergency. Hence study was aborted in these patients. Out of 100 participants, indications for UEI were respiratory failure (type 1 or type 2 with an inability to tolerate non-invasive ventilation), respiratory distress (respiratory rate of more than 34 per minute or/and usage of accessory muscles), airway protection for threatened airway (GCS < 8), circulatory collapse in form of refractory shock, and severe metabolic acidosis.

**Table 1:** Baseline characteristics of the study population

**Parameters**	**Values**
Age (Years), Mean ± SD	50.56 ± 14.44
Systolic BP (mmHg), Median (IQR)	120 (100.00–132.50)
Diastolic BP (mmHg), Median (IQR)	70 (60.00–88.00)
Heart Rate (BPM), Mean ± SD	98.47 ± 18.92
Respiratory Rate (CPM), Median (IQR)	24 (20.00–28.50)
Spo2 (%), Median (IQR)	93 (86.50–96.00)
RBS (mg/dL), Median (IQR)	141 (115–179)
Time since Solid Meal (hr) (*n* = 54), Median (IQR)	8.00 (6.00–12.00)
Time Since Liquid Meal (hr) (*n* = 46), Median (IQR)	9.50 (5.00–24.00)
**Parameters**	**Frequency (Percentage)**
Male	67 (67.0%)
Female	33 (33.0%)
Trauma	4 (4.0%)
Non-Trauma	96 (96.0%)
**Comorbidities**	
Diabetes mellitus (DM)	25 (23.4%)
Hypertension (HTN)	9 (8.4%)
Chronic obstructive pulmonary disease	9 (8.4%)
Chronic liver disease (CLD)	7 (6.5%)
Chronic kidney disease (CKD)	5 (4.7%)
Tuberculosis (TB)	4 (3.7%)
Coronary artery disease (CAD)	3 (2.8%)
Malignancy	2 (1.9%)
Interstitial lung disease (ILD)	1 (0.9%)
Hypothyroidism	1 (0.9%)
None	41 (38.3%)
**Indication for intubation**	
Low GCS (< 8)	53 (53%)
Respiratory failure (T1RF or T2RF)	22 (22%)
Respiratory distress (RR > 34)	16 (16%)
Circulatory collapse	5 (5%)
Metabolic acidosis	4 (4%)
**Induction Agent**	
Ketamine	61 (61%)
Etomidate	33 (33%)
Propofol	6 (6%)
**Number of attempts**	
Single Attempt	94 (94.0%)
Multiple Attempt	6 (6.0%)
**Aspiration**	
Present	8 (8%)
Absent	92 (92%)

The different ultrasound findings are represented in Table 2. Sixty six patients had an empty gastric antral status while 34 had distended antrum. The antral diameters were measured and gastric CSA and GV calculated. Out of 100 participants who underwent UEI, 8 participants had visible aspiration in the form of regurgitation of any stomach contents.

**Table 2:** Gastric ultrasound parameters

**USG GASTRIC STATUS**	**Frequency**
Empty (walls juxtaposed)	66 (66.0%)
Distended (rounded)	34 (34.0%)
**Antral Parameters**	**Median (IQR)**
USG CC Diameter (cm), Median (IQR)	1.60 (1.28–2.20)
USG AP Diameter (cm), Mean ± SD	4.16 ± 1.09
USG CSA (cm^2^), Median (IQR)	5.15 (3.25–8.32)
USG Gastric Volume (mL), Median (IQR)	29.65 (13.43–85.67)

### Time since solid meal (*n* = 54) and time since liquid meal (*n* = 46) to predict USG gastric status

In our study, the time since solid meal for empty gastric status ranged from 6–96 h (median of 11 h) and distended antrum ranged from 3–12 h (median of 6 h). The time since liquid meal for empty antrum ranged from 3–120 h (median was 10 h) and distended antrum ranged from 1–12 h (median was 4 h).

Time since solid meal was not normally distributed in the two subgroups of USG gastric status – empty vs distended. Thus, non-parametric test (Wilcoxon-Mann–Whitney U Test) was used to make group comparisons. There was a significant difference between the two groups in terms of time since solid meal (W = 657.50, *p* =  < 0.001), the median time being higher in the empty gastric status group. Strength of Association (Point-Biserial Correlation) is 0.44 (Large Effect Size).

The area under the ROC curve (AUROC) for time since solid meal for predicting gastric status was 0.903 (95%CI = 0.827–0.979), thus demonstrating excellent diagnostic performance. It was statistically significant (*p* =  < 0.001). A cut-off of time since solid meal ≤ 7.5 h predicts gastric status as distended with a sensitivity of 81% and a specificity of 86%.

The variable time since liquid meal was not normally distributed in the 2 subgroups of USG gastric status. Thus, non-parametric test (Wilcoxon-Mann–Whitney U Test) was used to make group comparisons. There was a significant difference between the two groups in terms of time since liquid meal (W = 259.000, *p* = 0.002), the median time being higher in the empty gastric status group. Strength of Association (Point-Biserial Correlation) is 0.28 (Medium Effect Size).

The AUROC for time since liquid meal for predicting gastric status was 0.852 (95%CI = 0.706–0.998), thus demonstrating good diagnostic performance. It was statistically significant (*p* = 0.002). A cut-off of time since liquid meal ≤ 6 h predicts gastric status as distended with a sensitivity of 88% and a specificity of 74%.

### Association between USG gastric status and aspiration

Fisher's exact test was used to explore the association between aspiration and gastric status as more than 20% of the total number of cells had an expected count of less than 5. There was a significant difference between the various groups in terms of distribution of gastric status (χ2 = 16.880, *p* =  < 0.001). Strength of association between the two variables (Cramer's V) is 0.41 (Moderate Association) indicating that a major proportion of patients who had aspiration had a distended gastric status.

### Time since solid meal (*n* = 54) and time since liquid meal (*n* = 46) to predict aspiration

The variables time since solid meal and time since liquid meal were not normally distributed in the two subgroups who had aspiration and who did not have aspiration. Thus, a non-parametric test (Wilcoxon-Mann–Whitney U Test) was used to make group comparisons. There was a significant difference between the two groups in terms of time since solid meal (W = 52.000, *p* = 0.036), the median time being highest in patients who had no aspiration. Strength of Association (Point-Biserial Correlation) is 0.15 (Small Effect Size). There was no significant difference between the two groups of aspiration regarding time since liquid meal (W = 32.500, *p* = 0.160).

The AUROC for time since solid meal for predicting aspiration was 0.788 (95%CI = 0.567–1), thus demonstrating fair diagnostic performance. It was statistically significant (*p* = 0.036). A cut-off of time since solid meal ≤ 6.5 h predict aspiration with a sensitivity of 80% and a specificity of 71%. The ROC analysis and diagnostic performance of time since the liquid meal was not statistically significant.

### Aspiration in terms of USG CC diameter

**Fig. 6:**
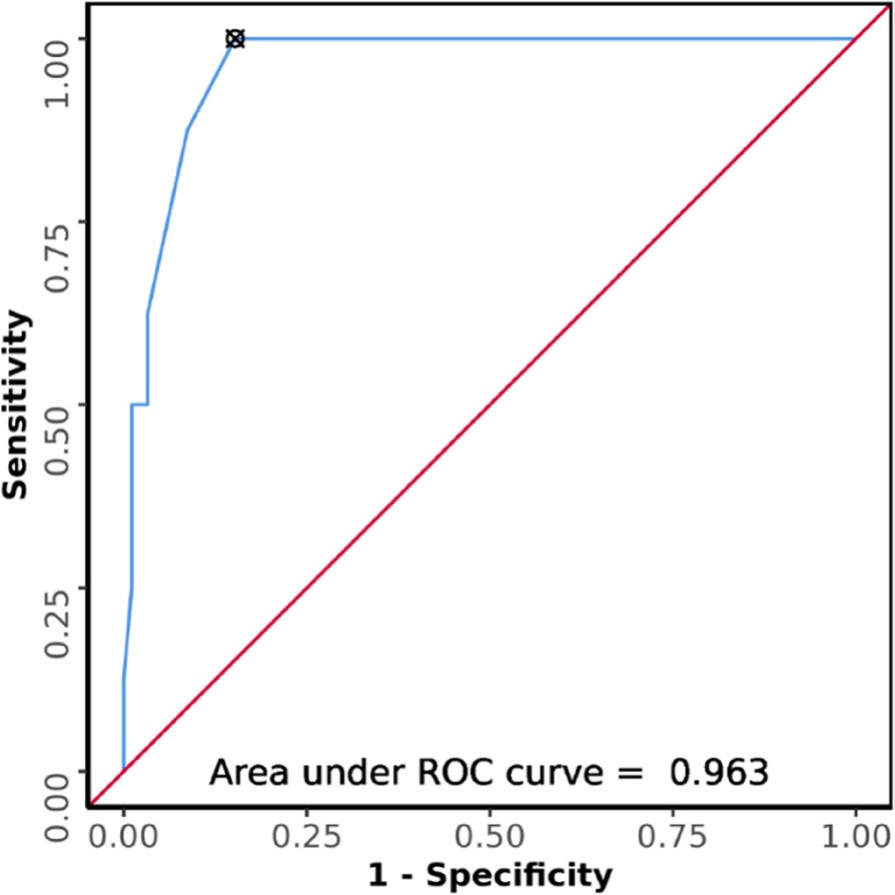
ROC curve showing diagnostic performance of USG CC diameter (cm) in predicting aspiration (*n* = 100)

The median (IQR) of CC diameter in the patients who aspirated was 2.53 cm (2.4 cm -2.73 cm). The median (IQR) of CC diameter in the patients who did not aspirate was 1.6 cm (1.2 cm – 2 cm). The variable CC diameter was not normally distributed in the two subgroups of aspiration. Non-parametric test (Wilcoxon-Mann–Whitney U Test) was used to make comparisons. There was a significant difference between the two groups in terms of CC diameter(cm) (W = 708.500, *p* =  < 0.001), with the median higher in those who had aspiration. Strength of Association (Point-Biserial Correlation) is 0.48 (Large Effect Size). The AUROC for CC diameter in predicting aspiration was 0.963 (95%CI = 0.925–1), demonstrating excellent diagnostic performance (Fig. 6). It was statistically significant (*p* =  < 0.001). A cut-off of CC diameter ≥ 2.35 cm predicts aspiration with a sensitivity of 88% and a specificity of 91%.

### Aspiration in terms of USG AP diameter

**Fig. 7:**
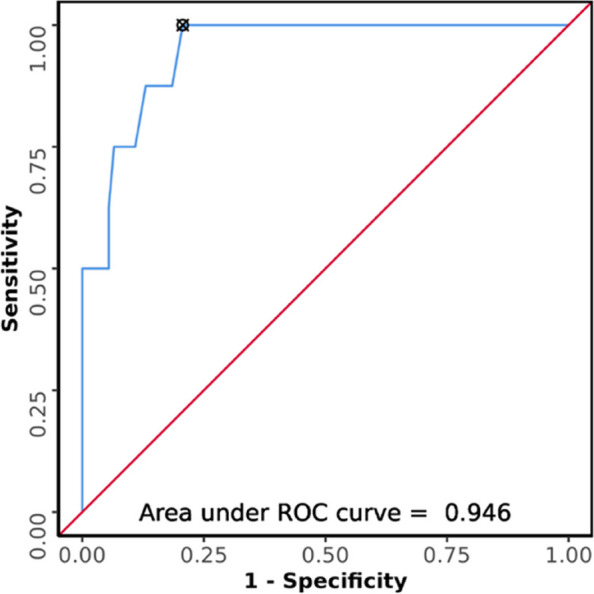
ROC curve analysis showing diagnostic performance of USG AP diameter (cm) in predicting aspiration (*n* = 100)

The median (IQR) of AP diameter in the patients who aspirated was 6.05 cm (5.5 cm -6.58 cm). The median (IQR) of AP diameter in the patients who did not aspirate was 3.95 cm (3.27 cm—4.6 cm). The variable AP diameter was not normally distributed in the two subgroups of aspiration. Non-parametric test (Wilcoxon-Mann–Whitney U Test) was used to make group comparisons. There was a significant difference between the two groups in terms of AP diameter (W = 696.500, *p* =  < 0.001), with the median AP diameter being highest in those who aspirated. Strength of Association (Point-Biserial Correlation) is 0.5 (Large Effect Size). The AUROC for AP diameter for predicting aspiration was 0.946 (95%CI = 0.89–1), demonstrating excellent diagnostic performance (Fig. 7). It was statistically significant (*p* =  < 0.001). A cut-off of AP diameter ≥ 5.15 cm predicts aspiration with a sensitivity of 88% and a specificity of 87%.

### Aspiration in terms of USG CSA

The median of USG CSA in the patients who aspirated was 12.55cm^2^ (IQR: 10.28cm^2^ -13.86cm^2^), and in those who did not aspirate was 4.84cm^2^ (3.04cm^2^—7.39cm^2^). USG CSA was not normally distributed in the two subgroups of aspiration. Hence, Wilcoxon-Mann–Whitney U Test was used to make group comparisons. There was a significant difference between the two groups in terms of CSA (W = 708.500, *p* =  < 0.001), with the median more in the participants who had aspiration. Strength of Association (Point-Biserial Correlation) is 0.57 (Large Effect Size).

**Fig. 8:**
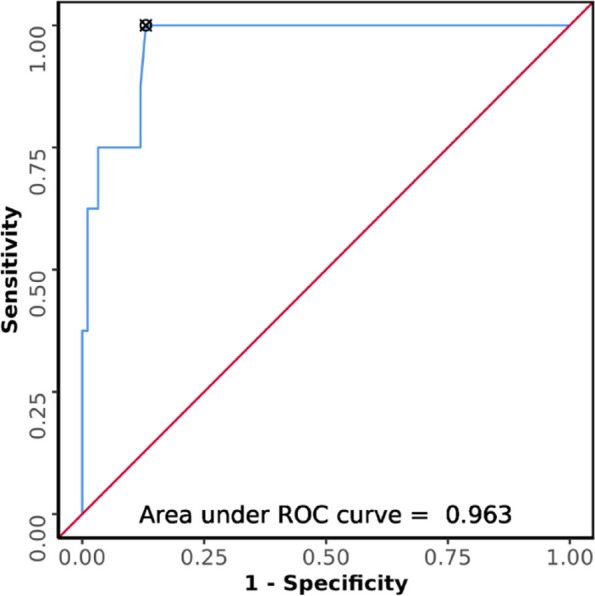
ROC curve analysis showing diagnostic performance of USG: CSA (cm.^2^) in predicting aspiration (*n* = 100)

The AUROC for CSA for predicting aspiration was 0.963 (95%CI = 0.92–1), demonstrating excellent diagnostic performance (Fig. 8). It was statistically significant (*p* =  < 0.001). A cut-off of USG CSA ≥ 9.27cm^2^ predicts aspiration with a sensitivity of 100% and a specificity of 87%.

### Aspiration in terms of USG gastric volume

The median of GV in those who aspirated was 146.37 mL (IQR: 120.1 mL-177.4 mL), and for those who did not aspirate was 26.82 mL (IQR: 12.09 mL-58.03 mL). GV was not normally distributed in the two subgroups of aspiration. Wilcoxon-Mann–Whitney U Test was used to make group comparisons. There was a significant difference between the two groups in terms of GV (W = 721.000, *p* =  < 0.001), with the median higher in those who aspirated. Strength of Association (Point-Biserial Correlation) is 0.62 (Large Effect Size).

**Fig. 9:**
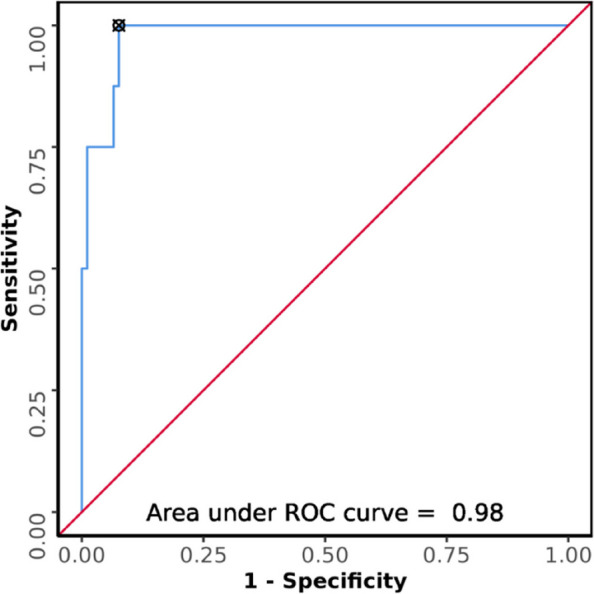
ROC curve analysis showing diagnostic performance of USG gastric volume (mL) in predicting aspiration (*n* = 100)

The AUROC for GV in predicting aspiration was 0.98 (95%CI = 0.953–1), demonstrating excellent diagnostic performance (Fig. 9). It was statistically significant (*p* =  < 0.001). A cut-off of Gastric Volume ≥ 111.594 mL predicts aspiration with a sensitivity of 100% and a specificity of 92%.

Comparison of the diagnostic performance of different variables in predicting aspiration is summarized in Table 3.

**Table 3:** Comparison of the diagnostic performance of various predictors in predicting aspiration

Predictor	Value of Presictor	AUROC	95% CI	P	Sn	Sp	PPV	NPV	DA
Time Since Liquid Meal (Hr)	-	0.748	0.367–1	0.160	-	-	-	-	-
Time Since Solid Meal (Hr)	≤ 6.5 h	0.788	0.567–1	0.036	80%	71%	22%	97%	72%
USG: AP Diameter (cm)	≥ 5.15 cm	0.946	0.89–1	< 0.001	88%	87%	37%	99%	87%
USG: CC Diameter (cm)	≥ 2.35 cm	0.963	0.925–1	< 0.001	88%	91%	47%	99%	91%
USG: CSA (cm^2^)	≥ 9.27 cm^2^	0.963	0.92–1	< 0.001	100%	87%	40%	100%	88%
USG: Gastric Volume (mL)	≥ 111.594 mL	0.980	0.953–1	< 0.001	100%	92%	53%	100%	93%

## Discussion

Patients who required UEI in the emergency department were included in the study. The main indications for UEI were acute respiratory failure (either fault in the oxygenation or hypercapnia associated with an inability to tolerate non-invasive ventilation), respiratory distress (with a respiratory rate of more than 34 per minute or/and usage of accessory muscles), airway protection for threatened airway, circulatory collapse, and severe metabolic acidosis. The indications were similar to a study conducted by Koenig et al. who did GUS on emergency patients before UEI [29]. We did no intervention to remove the gastric content because this would further delay intubation. Also, any attempt to pass a nasogastric or orogastric tube might provoke regurgitation by the gag reflex. Thus, standard practice as per the patient assessment and institutional protocol was adhered to, while ultrasound measurements were recorded.

Our study found no significant association between aspiration and age, gender, or vasopressor use. Association of aspiration with comorbidities was not significant (*p* > 0.05) probably due to the small number of participants who had aspiration (*n* = 8), and hence no meaningful statistical comparison could be made with each comorbidity. There was more occurrence of aspiration in trauma patients when compared to non-trauma cases. D.J Lockey et al. also found that 38% of patients with severe trauma who required surgery had a high prevalence of aspiration as a perioperative complication [31].

We found the median of gastric CSA of an empty antrum was 3.87cm^2^ (IQR: 2.83cm^2^–5.12cm^2^) and a distended antrum was 9.43cm^2^ (IQR: 8.03cm^2^–10.5cm^2^). The French team led by Dr.Bouvet reported gastric CSA measured in the semi-recumbent position of > 3.4cm^2^ in non-pregnant patients correlated with a non-empty stomach [17]. While Perlas described an empty antrum would be approximately equal to the thickness of the gastric wall (approximately 2–5cm^2^) [22]. We found the median USG gastric volume of an empty antrum was 17.83 mL (IQR: 4.04 mL–28.87 mL), and a distended one was 109.24 mL (IQR: 82.39 mL–118.95 mL).

Those who had a distended antrum made a larger proportion of participants who had a visible aspiration. According to Richa et al., only 14 patients out of 100 ESRD patients had an empty stomach (Perla's grade 0) 6 h after a light meal [32]. We found time since solid meal ≤ 7.5 h predict USG gastric state as distended with a sensitivity of 81%, and a specificity of 86%. Time since liquid meal ≤ 6 h predicts USG gastric status as distended with a sensitivity of 88% and a specificity of 74%. Van de Putte(2017) mentioned that 3–5% of fasted individuals could also have a distended stomach with a volume > 1.5 mL/kg [33].

ROC curve analysis of different parameters were used for the prediction of aspiration using cut-off values. The ROC analysis and diagnostic performance of time since the liquid meal was not statistically significant. Hence, time since a liquid meal is not a good predictor for aspiration. A cut-off of time since solid meal ≤ 6.5 h predict aspiration with a sensitivity of 80% and a specificity of 71%.

A cut-off of USG CC diameter ≥ 2.35 cm predicts aspiration with a sensitivity of 88% and a specificity of 91%, while a cut-off of USG AP diameter ≥ 5.15 cm predicts it with a sensitivity of 88% and a specificity of 87%. Sharma et al. suggested that the CC diameter can be a simple surrogate of the residual gastric volume. They found the CC diameter increased linearly with increasing gastric residual volume measured by aspirated tube feed. A CC diameter of < 10 cm predicted a gastric volume of < 500 mL [34].

According to previous studies, a cut-off value of antral CSA of 3.4 cm^2^ was labeled as high risk for aspiration and they tend to have a GV greater than 0.8 mL/kg with a sensitivity of 91% and a specificity of 71% [35]. We report the median of GV in the patients who aspirated was 146.37 mL, and it ranged from 111.59 mL-201.01 mL. The median of GV in those who did not aspirate was 26.82 mL, and it ranged from 0 mL-131.2 mL.

In our study, USG CSA cut-off ≥ 9.27 cm^2^ predicts aspiration with a sensitivity of 100% and a specificity of 87% while the USG GV ≥ 111.594 mL predicts aspiration with a sensitivity of 100% and a specificity of 92%. Van de Putte and Perlas(2014) stated that volumes up to 1.5 mL/kg approximating to 100-110 mL, are considered normal and safe [9]. The values above this could cause clinically significant aspiration. This GV would correlate to an antral CSA between 9cm^2^ and 10cm^2^ measured in the right lateral decubitus position according to the Perlas formula [28]. However, this threshold is for the Caucasian ethnicity. Indian adults are built differently and have lower average weight. Hence, a lower gastric volume should be considered safer.

Participants who were induced with ketamine had a lesser incidence of aspiration, while participants induced with etomidate and propofol had more incidence of aspiration. This difference was statistically significant in the study (χ2 = 4.753, *p* = 0.047). Ketamine preserves the pharyngeal and laryngeal reflexes. Since we took only visible aspiration into account, micro-aspiration due to secretions could have been missed [36]. Nevertheless, this paves the way for more research on this topic. The number of attempts for intubation caused no significant difference in aspiration (χ2 = 5.566, *p* = 0.072).

We have to remember that any cut-off value is not considered foolproof. The sensitivity and specificity will change in reverse directions when the values are higher and lower. The NPV of the test parameters (USG CC, AP, CSA, and GV) is the most important because the correct diagnosis of an empty stomach is of higher priority during emergency airway management [28]. We considered the NPV and sensitivity the most critical parameters for deciding the cut-off since we are more concerned about false negatives.

We found that GUS is feasible to find the prandial status of patients before undergoing UEI in the emergency rooms. It can be done quickly without affecting routine patient care. We present threshold gastric antral parameters that can be used to predict aspiration along with its diagnostic accuracy. This can help the treating ED physician take adequate precautions, decide on intubation techniques and treatment modifications to aid in better patient management. We found that USG parameters are a better tool for predicting aspiration when compared to the history of the last meal and nil per oral status in view of the diagnostic accuracy. Any means to take adequate precautions and to prevent aspiration is of utmost importance as it can cause drastic consequences increasing both morbidity and mortality.

## Limitations

1. Healthy people without any illness, patients with altered gastric anatomy, pregnant population and children were not part of the study. Thus, this study lacks information about the study parameters on this population.
2. Micro-aspirations and follow-up development of consolidative patch in chest x-ray were not accounted for in the study. Only visible aspirations were considered, which would need larger gastric volume.
3. Quantifying a distended stomach with solid content may not be accurate as the posterior wall of the gastric antrum would be obscured in the presence of solid contents.
4. The number of patients with each comorbidity was limited as well as overlapping; thus, extrapolating the association for risk of aspiration with each comorbidity was not statistically significant.
5. The weight and BMI of the patient were not taken into consideration, and hence the exact threshold of gastric volume per kg could not be calculated.
6. Even though trauma patients have more chance of having a full stomach and aspiration, the number of trauma patients included in the study was less.

## Conclusion

Point of care gastric ultrasound is a useful non-invasive bedside tool for risk stratification for aspiration. It is feasible and accurate in the busy emergency rooms to predict aspiration in patients requiring UEI. We present a threshold gastric volume and other gastric parameters (CC and AP diameters, CSA, GV) that can be used to predict aspiration along with its diagnostic accuracy. This can help the treating ED physician in deciding intubation techniques and treatment modifications and aid in further patient management. Larger prospective RCT with participants in two limbs – one with GUS assessment and one with no GUS assessment investigating the incidence of aspiration and appropriate intervention to reduce the same would be necessary to conclusively verify the clinical benefits of using bedside GUS before intubation.

## Data Availability

The datasets used and/or analysed during the current study are available from the corresponding author on reasonable request.

## Abbreviations

ED: Emergency department
GV: Gastric volume
ASA: American Society of Anesthesiologists
NPO: Nil per oral
USG: Ultrasonography
GUS: Gastric ultrasound
PoCUS: Point-of-care ultrasound
CSA: Cross-sectional area
RLD: Right lateral decubitus
AP: Anteroposterior
CC: Craniocaudal
BMI: Body mass index
PPV: Positive predictive values
NPV: Negative predictive values
UEI: Urgent emergency intubation
LUQ: Left upper quadrant
MRI: Magnetic resonance imaging
CT: Computed tomography
RSI: Rapid sequence induction
ROC: Receiver operating characteristic
AUROC: Area under the ROC curve
DM: Diabetes mellitus
HTN: Hypertension
COPD: Chronic obstructive pulmonary disease
CLD: Chronic liver disease
CKD: Chronic kidney disease
TB: Tuberculosis
CAD: Coronary artery disease
ILD: Interstitial lung disease
GCS: Glasgow-coma-scale
